# Recent Advances in DMSO-Based Direct Synthesis of Heterocycles

**DOI:** 10.3390/molecules27238480

**Published:** 2022-12-02

**Authors:** Hai-Lei Cui

**Affiliations:** Laboratory of Asymmetric Synthesis, Chongqing University of Arts and Sciences, 319 Honghe Ave., Yongchuan, Chongqing 402160, China; cuihailei616@163.com

**Keywords:** cyclization, dimethyl sulfoxide, heterocycle, Kornblum oxidation, methyl(methylene)sulfonium

## Abstract

Besides serving as a low-toxicity, inexpensive and easily accessible solvent, dimethyl sulfoxide (DMSO) has also been extensively used as a versatile reagent for the synthesis of functionalized molecules. Dimethyl sulfoxide can not only be utilized as a carbon source, a sulfur source and an oxygen source, but also be employed as a crucial oxidant enabling various transformations. The past decade has witnessed a large number of impressive achievements on the direct synthesis of heterocycles as well as modifications of heterocyclic compounds by applying DMSO as a reagent. This review summarized the DMSO-based direct heterocycle constructions from 2012 to 2022.

## 1. Introduction

Dimethyl sulfoxide (DMSO) has long been used as a low-toxicity, inexpensive and easily accessible solvent [1]. Furthermore, dimethyl sulfoxide can also be applied as a versatile reagent for the construction of functionalized molecules. Several powerful DMSO-based transformations, such as Swern oxidation (ClCOCOCl/DMSO), Moffatt oxidation (DCC/HX/DMSO), Parikn–Doering oxidation (SO_3_/DMSO), Kornblum oxidation (RX/DMSO), Corey–Chaykovsky reaction (MeI/DMSO) and Pumerer rearrangement (Ac_2_O/sulfoxide), have been frequently utilized in organic synthesis [2,3,4,5,6,7]. Dimethyl sulfoxide is a valuable synthon for constructing heterocyclic compounds by acting as a carbon source, sulfur source and oxygen source as well as a key oxidant for various transformations. A large number of efficient methodologies have been developed in the past decades for constructing functionalized heterocycles, either by the direct synthesis manner or by the late-stage modification route.

**Figure 1:**
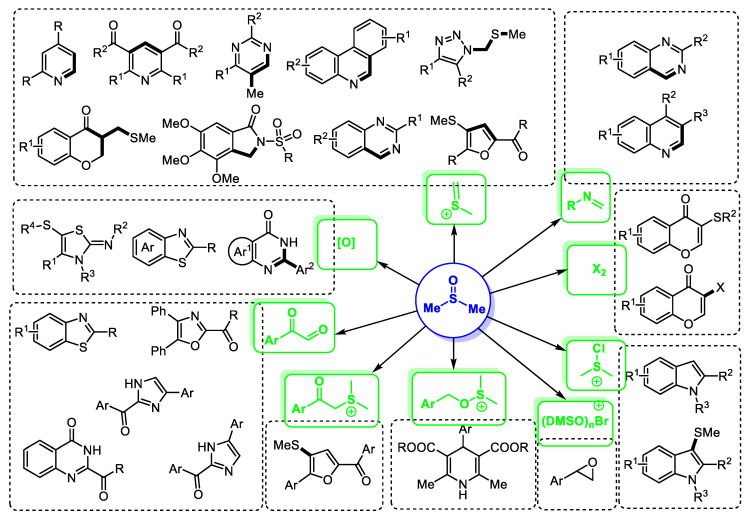
DMSO-based direct synthesis of heterocycles.

DMSO-based reaction systems feature good flexibility in designing transformations, a diversity of intermediates and compatibility of functional groups. For example, as shown in Figure 1, there are many common intermediates that can be formed in situ in the DMSO-involved reaction systems, including methyl(methylene)sulfonium, chlorodimethylsulfonium, bromodimethylsulfonium, molecular bromine and arylglyoxal. These active species could be captured directly or further converted into other reactive intermediates. Furthermore, other active species such as dimethyl sulfur ylide and imine can also be formed efficiently for assembling heterocycles. Additionally, DMSO can be employed as an oxidizing agent for the construction of heterocyclic molecules through the oxidative formation of key intermediates or products.

In 2016, the groups of Wu and Magolan respectively systematically summarized and discussed the reactions using dimethyl sulfoxide as a synthon [8,9]. Subsequently, Mahdavi and coworkers have well reviewed and classified the methodologies with DMSO as a reagent for organic synthesis [10]. In addition, Procter and Maulide recorded the developments of transforming sulfoxides, which can offer a good inspiration for exploring sulfoxide chemistry [11,12]. However, there is no special summary focusing on the DMSO-based heterocycle synthesis [13]. In order to further extend the applications of DMSO as a synthon for heterocycle synthesis and to fully clarify the chemistry of DMSO, it is very useful to document the achievements in the field of DMSO-based heterocycle synthesis. This review summarized the direct DMSO-based heterocycle constructions from 2012 to 2022. The review highlighted the unique roles of DMSO in heterocycle synthesis and showcased some representative reaction pathways. The emphasis on DMSO-derived active intermediates and mechanistic pathways may be beneficial for understanding the chemistry of DMSO and the stepwise formation of heterocycles. Since the use of many DMSO-involved active species and the design of reaction pathways can be further rationally expanded, this review may provide a source of inspiration for designing novel architectures and protocols for heterocycle synthesis.

It should be mentioned that this review only discussed the reports using acyclic starting materials for heterocycle synthesis. The methods involving direct modifications of already established heterocyclic architectures will not be included in the review [14]. To clearly understand the role of DMSO in these protocols, this review was divided into several categories, including DMSO as the source of CH, DMSO as the source of CH_2_, DMSO as the source of quaternary C, DMSO as the source of CH and CH_3_, DMSO as the source of SCH_3_, DMSO as the source of CH_2_SCH_3_, DMSO as the source of CH_2_ and CH_2_SCH_3_, DMSO as the source of O, DMSO as the oxidant, and DMSO-based heterocycle synthesis through halogenation. The reactions using DMSO as the source of CH were further subdivided by the types of synthesized heterocycles such as pyridines, quinolines, phenanthridines, pyrimidines, quinazolines, benzothiazoles, benzimidazoles, and furans.

## 2. DMSO as the Source of CH

A series of heterocycles such as pyridines, quinolines, phenanthridines, pyrimidines, quinazolines, benzothiazoles, benzimidazoles, and furans can be constructed directly using dimethyl sulfoxide as the source of C–H. The C–H moiety was always incorporated into aromatic molecules. Thus, aromatization was involved in most cases. Among these approaches, methyl(methylene)sulfonium formed through Pummerer-type rearrangement and imines generated via the elimination of amino thioethers are common active species.

### 2.1. Synthesis of Pyridine

**Scheme 1:**
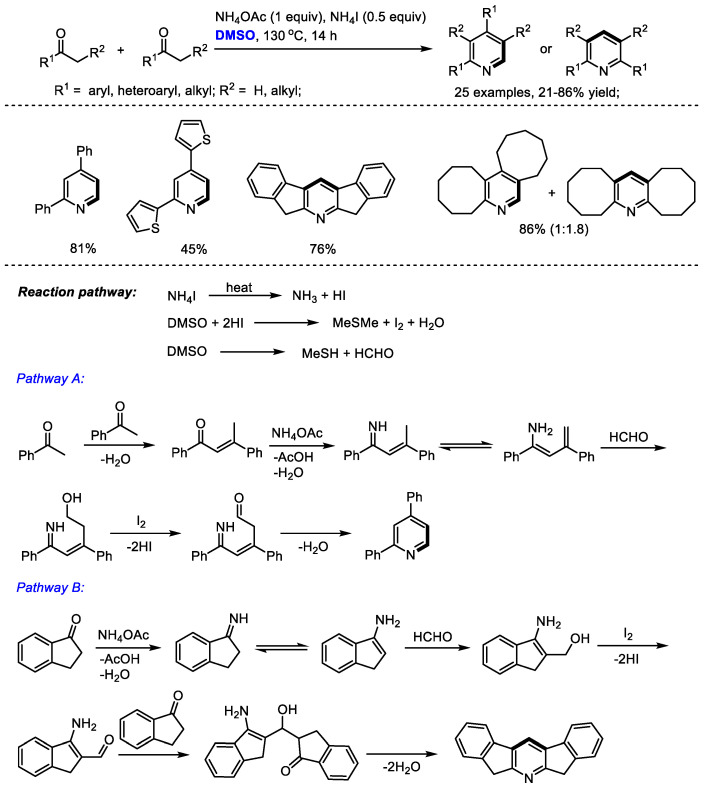
NH_4_I-mediated synthesis of pyridines [15].

Pyridine derivatives have been found frequently in organocatalysts, ligands, natural products, pharmaceuticals and other important molecules. Therefore, the direct synthesis of substituted pyridines has attracted considerable interest. Yuan and coworkers reported an ammonium iodide-mediated synthesis of substituted pyridines with ketones, dimethyl sulfoxide and ammonium acetate in 2015 (Scheme 1) [15]. The C–H moiety can be incorporated at the C2 and C4 positions of pyridine. Interestingly, in the cases using methyl ketones, unsymmetrical pyridines were isolated as the sole products (Pathway A); while the use of non-methyl ketones yielded unpredictable products including only symmetrical or nonsymmetrical products, or a mixture of the two (Pathways B and A). An aldol reaction of dienamine intermediate with formaldehyde occurred in the cases through Pathway A; while an aldol addition of enamine and formaldehyde was involved in Pathway B. A range of substituted pyridines bearing aryl and alkyl groups can be prepared with high efficiency from easily accessible starting materials (25 examples, up to 86% yield).

**Scheme 2:**
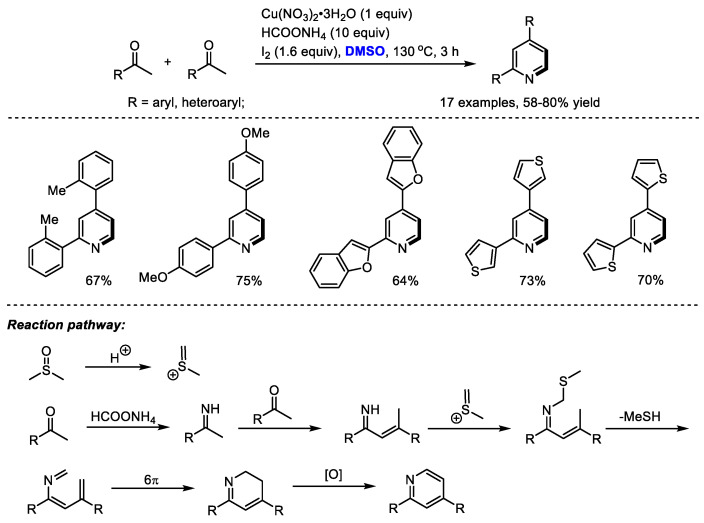
I_2_/DMSO-promoted synthesis of pyridines [16].

The I_2_/DMSO reaction system can also be used for synthesizing pyridines with high efficiency. Wu and Gao constructed a variety of substituted pyridines with aryl and heteroaryl ketones (Scheme 2) [16]. The authors proposed a Claisen condensation, methylthiomethylenation, C–S cleavage, 6-π electrocyclization and aromatization cascade sequence. This process features a wide substrate scope, good functional group compatibility, excellent regioselectivity and high efficiency. The current methodology represents a novel strategy for the capture of the in situ-formed methyl(methylene)sulfonium intermediate from dimethyl sulfoxide.

**Scheme 3:**
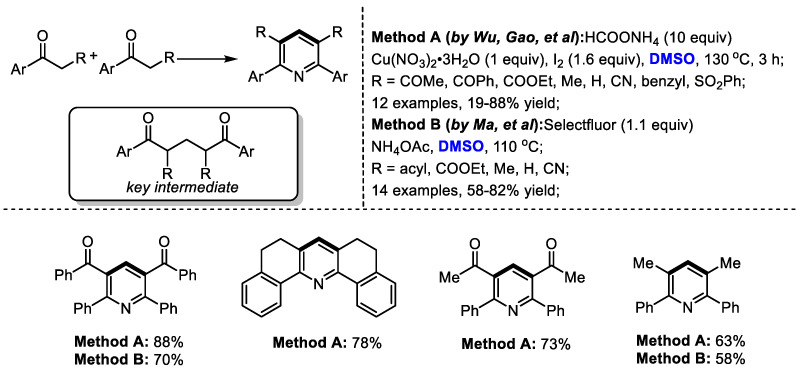
Introduction of C–H moiety at the C4 position of pyridines [16,17].

Interestingly, α-substituted aryl ketones would undergo a different reaction pathway under the same reaction system. Wu and Gao found that when submitting α-substituted aryl ketones to their HCOONH_4_/I_2_/Cu(NO_3_)_2_/DMSO reaction system, a series of functionalized pyridines were yielded by forming methylene-bridged 1,5-diketone as a key intermediate (Scheme 3). In contrast, differently from the reactions using methyl ketones, C–H moiety was introduced at the C4 position of the pyridine. Subsequently, the Ma group also synthesized polysubstituted pyridines using a selectfluor/DMSO reaction system [17]. Selectfluor was used as the activator of DMSO. DMSO served as the methine source for both processes.

**Scheme 4:**
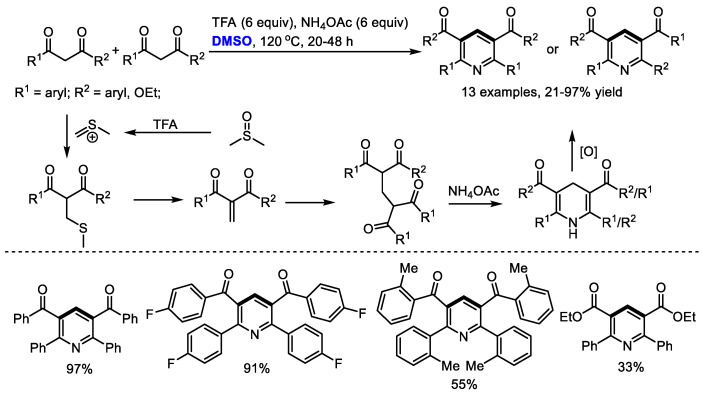
Synthesis of pyridines in the presence of TFA/DMSO [18].

Bronsted acids such as trifluoroacetic acid (TFA) can be used as the activators of DMSO for the methylenation of 1,3-dicarbonyl compounds. Annulation of in situ-generated methylene-bridged dicarbonyl compounds could offer substituted pyridines. In 2017, Cui and Cheng also employed DMSO as the source of methine for the synthesis of Hantzsch-type pyridines through a three-component reaction of 1,3-dicarbonyl compounds, DMSO and ammonium salts (13 examples, up to 97% yield). A transition-metal free oxidative methylenation was proposed as the key step and methylene-bridged bis-1,3-dicarbonyl compound was suggested as the key intermediate. It should be noted that the methylene-bridged bis-1,3-dicarbonyl compounds could be readily prepared through an acetic acid mediated reaction of 1,3-dicarbonyl compounds and dimethyl sulfoxide in moderate to excellent yields (Scheme 4) [18].

**Scheme 5:**
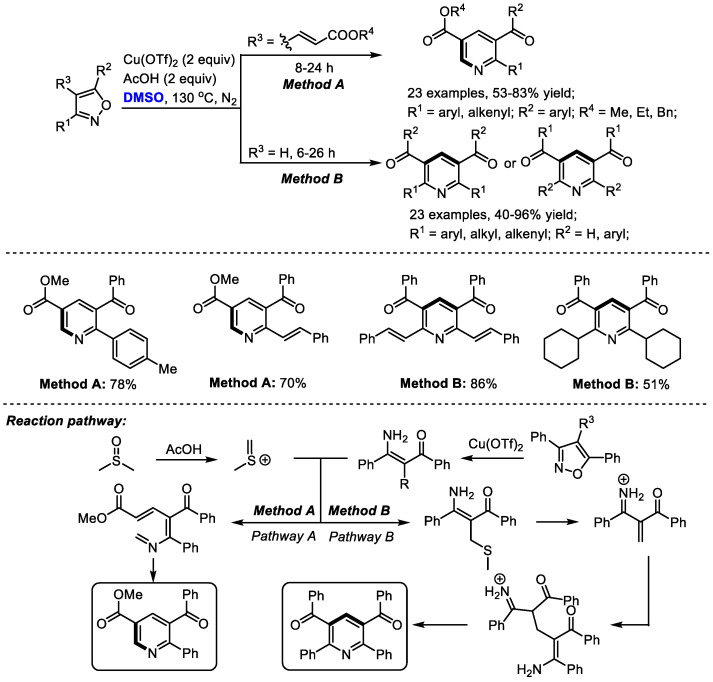
Synthesis of pyridines with isoxazoles [19].

In situ -generated enaminones could be used as effective precursors instead of the direct use of ketone or 1,3-dicarbonyl compound for pyridine synthesis. The Kapur group explored the unusual reactivity of 4-vinyl isoxazoles in the copper salts-promoted construction of pyridines in 2020. Isoxazoles have always been used as versatile intermediates for constructing pharmaceutically important heterocycles (Scheme 5) [19]. Particularly, 3,5-diaryl isoxazoles have been employed as masked enaminones as *C*,*N*-dinucleophilic synthons to couple with various building blocks, yielding structurally diverse heterocyclic molecules. 

In this study, isoxazole converted into the corresponding enaminone in the presence of copper salts. Meanwhile, methyl(methylene)sulfonium was formed from DMSO in the presence of acid as the activator. The reaction of enaminone with methyl(methylene)sulfonium may produce a conjugated imine for further electrocyclization when using alkenylated substrates (Pathway A). On the other hand, α,β-unsaturated dicarbonyls may be generated to undergo Michael addition, giving methylene-bridged 1,3-dicarbonyl compounds. Further intramolecular 1,4-addition delivered a Hantzsch pyridine intermediate and a final aromatization-yielded tetrasubstituted pyridine. It was found that much lower yield was obtained in the case with a catalytic quantity of copper salts, indicating that the copper salts may also serve as an oxidant. 

A wide range of aryl and alkenyl groups can be introduced into the pyridine skeleton delivering densely substituted pyridines (Method A, 23 examples, 53–83% yield; Method B, 23 examples, 40–96% yield). Dimethyl sulfoxide was employed as a one-carbon surrogate in this nicotinate synthesis.

### 2.2. Synthesis of Quinoline

**Scheme 6:**
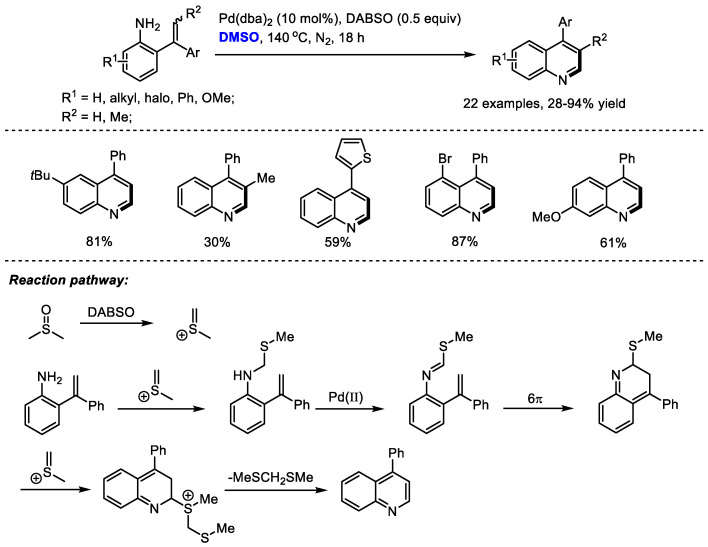
Pd-catalyzed synthesis of quinolines [20].

As a ubiquitous structural motif present in a large number of natural products and pharmaceutically important molecules, the efficient synthesis of functionalized quinolines has drawn much attention. Cheng and coworkers prepared a variety of 4-aryl quinolines via palladium-catalyzed carbonannulation of *ortho*-vinylanilines and dimethyl sulfoxide in 2017 (Scheme 6) [20]. By in situ formation of imine from DMSO and primary amine, C–H moiety can be introduced at the C2 position of the quinoline. 1,4-Diazabicyclo [2.2.2] octane bis(sulfur dioxide) adduct (DABSO) was applied as the activator of DMSO. The methine fragment was smoothly incorporated into the quinoline skeleton in the presence of DABSO, providing 4-substituted quinolines in moderate to good yields (22 examples, 28–94% yield). 

The authors proposed that methyl(methylene)sulfonium cation can be generated in situ by activating DMSO with DABSO. A *N*-methylthiomethylenation, palladium-catalyzed oxidation, electrocyclization and a final aromatization by the elimination of bis(methylthio)methane delivered the desired quinolines.

**Scheme 7:**
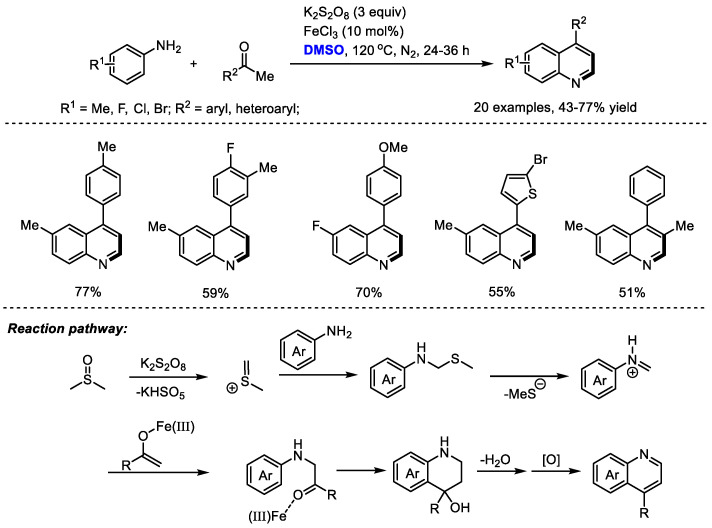
K_2_S_2_O_8_-mediated synthesis of quinolines [21].

In 2017, the Singh group developed a K_2_S_2_O_8_-mediated oxidative annulation of anilines, aryl ketones and dimethyl sulfoxide for the efficient assembly of 4-arylquinolines (Scheme 7) [21]. K_2_S_2_O_8_ was employed as the activator of DMSO and DMSO acted as a methine equivalent. Both anilines and aryl ketones displayed wide substrate scopes.

It was found that FeCl_3_ has a more pronounced effect on the reactions using electron-rich acetophenone. No influence on the reaction outcome was observed when adding FeCl_3_ to the reactions with acetophenone bearing electron-withdrawing fluorine and the nitro group. These observations suggested that FeCl_3_ may be helpful for the enolization of acetophenones. The authors proposed that a key iminium may be formed from DMSO and aniline, which would undergo a Mannich addition with iron-activated aryl ketone, annulation, dehydration and final aromatization to yield the quinoline product.

**Scheme 8:**
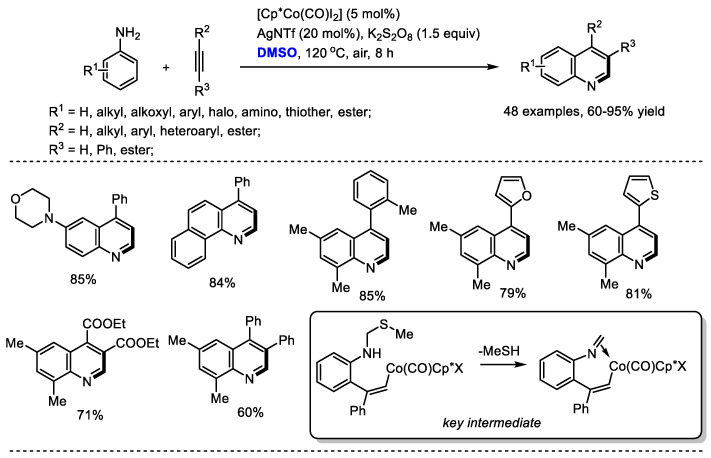
Co(III)-catalyzed synthesis of quinolines [22].

Substituted alkynes can be used instead of ketones as reaction partners to react with the in situ-generated imine for quinoline synthesis. Yi and Zhang reported a direct Co(III)-catalyzed assembly of quinoline with dimethyl sulfoxide, anilines and alkynes (48 examples, up to 95% yield). K_2_S_2_O_8_ was utilized as the activator of DMSO for generating methyl(methylene)sulfonium cation. A mechanistic study suggested that this transformation was initiated with C–H activation and 2-vinylbenzenamine species may be the active intermediate for this process. It is worthy of note that the reaction has several advantages including the utilization of simple and easily accessible materials, privileged framework-containing products, exclusive regioselectivity, good functional group tolerance and a wide substrate scope (Scheme 8) [22].

### 2.3. Synthesis of Phenanthridine

**Scheme 9:**
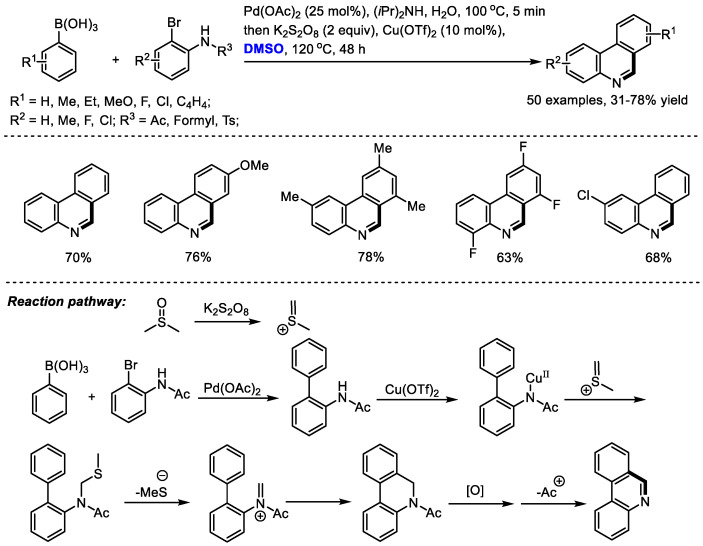
Pd-catalyzed synthesis of phenanthridines [23].

Phenanthridine can be found as a core substructure in a series of biologically active compounds including Trispheridine, Decarine, Norchelerythrine, Avicine, Nitidine and Fagaronine. Ma and coworkers developed a one-pot synthesis of phenanthridines with arylboronic acids and *o*-bromo arylamides through a Suzuki coupling/*N*-methylthiomethylenation/elimination/electrocyclization/aromatization cascade sequence (50 examples, 31–78% yield). Pd(OAc)_2_ was employed to catalyze the Suzuki reaction delivering biaryls. K_2_S_2_O_8_ was applied as the activator of DMSO to generate meth(methylene)sulfonium, which was used for the formation of a third cycle in the presence of a copper catalyst (Scheme 9) [23]. The electrocyclization of in situ-formed imine could be an efficient way of constructing fused rings.

### 2.4. Synthesis of Pyrimidine

**Scheme 10:**
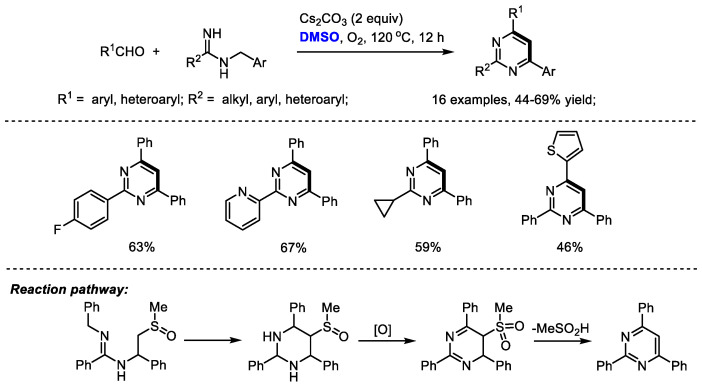
DMSO-involved synthesis of pyrimidines [24].

Amidine is an attractive nitrogen source for pyrimidine and quinazoline synthesis under a DMSO-involved reaction system. Cheng and coworkers synthesized a range of 2,4,6-triaryl pyrimidines through a base-mediated [4 + 1 + 1] annulation of aldehydes, *N*-benzyl amidines and dimethyl sulfoxide. DMSO was activated by a base, rather than either Lewis acid or electrophile, serving as the methine source. Dioxygen was used as the sole eco-friendly oxidant in this process. Unfortunately, although aryl aldehydes can be compatible in this reaction, aliphatic aldehydes failed to yield corresponding pyrimidine. As proposed by the authors, a 5-(methylsulfinyl)-2,4,6-triarylhexahydropyrimidine may be generated as an intermediate which would be oxidized by molecular dioxygen and undergo elimination to yield the desired product (Scheme 10) [24].

### 2.5. Synthesis of Quinazoline

Quinazoline as a core-structure is widely distributed in natural products and synthetic bioactive compounds including protein tyrosine kinase inhibitors, anticancer agents, antiviral agents and antitubercular agents. In 2013, Zhang and Xiong disclosed an efficient copper-catalyzed synthesis of quinazolines with amidines and dimethyl sulfoxide (24 examples, 52–93% yields). Unfortunately, cyclic amidine substrates failed to yield corresponding quinazolines, demonstrating the limitation of this protocol. 

**Scheme 11:**
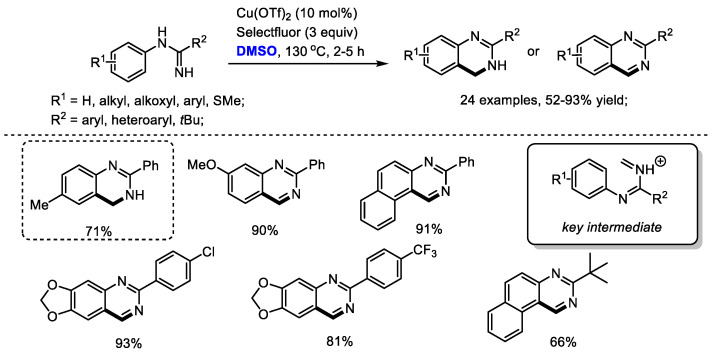
Cu-catalyzed synthesis of quinazolines [25].

On the basis of the results, an iminium was proposed as the key intermediate for this transformation, which would undergo further electrocyclization and electrophilic addition to produce dihydroquinazolines. Pleasingly, the authors also successfully employed DMF, DMA, TMEDA and NMP as the one-carbon synthon for constructing quinazolines (Scheme 11) [25].

**Scheme 12:**
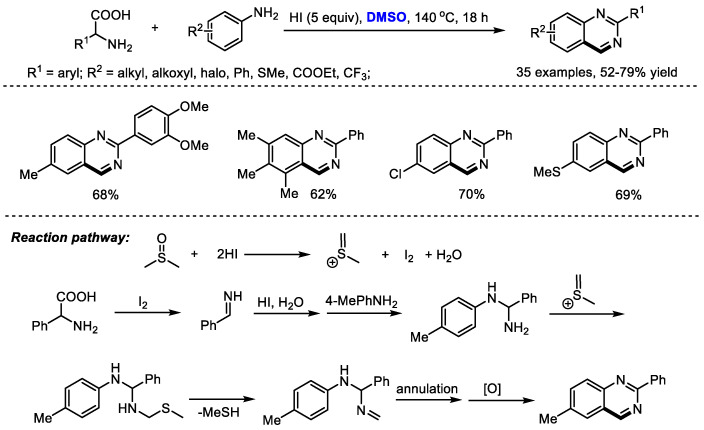
HI-promoted synthesis of quinazolines [26].

Amino acids as key building blocks have been widely used for preparing heterocyclic compounds through a variety of processes such as an Ugi reaction and a decarboxylation reaction. Wu and Wu synthesized substituted quinazolines with carboxylic acids, aromatic amines and dimethyl sulfoxide, through an HI-mediated amino acid catabolism/reconstruction together with the insertion/cyclization of DMSO (Scheme 12) [26]. In this process, amino acids were catabolized and served as a carbon and nitrogen source. Reconstruction with aromatic amines and subsequent insertion/cyclization with DMSO led to a large number of functionalized quinazolines (35 examples, 52–79% yield).

### 2.6. Synthesis of Benzothiazole

In the presence of a sulfur source, benzothiazole can be constructed efficiently under DMSO-involved protocols. Zhu, Liang and Yu achieved an efficient three-component synthesis of 2-unsubstituted benzothiazoles with *o*-iodoanilines, K_2_S and dimethyl sulfoxide (Method A). A series of *o*-iodoanilines bearing electron-donating or electron-withdrawing groups all can be compatible with this process, producing corresponding benzothiazoles in moderate to good yields (16 examples, 37–98% yield). 

**Scheme 13:**
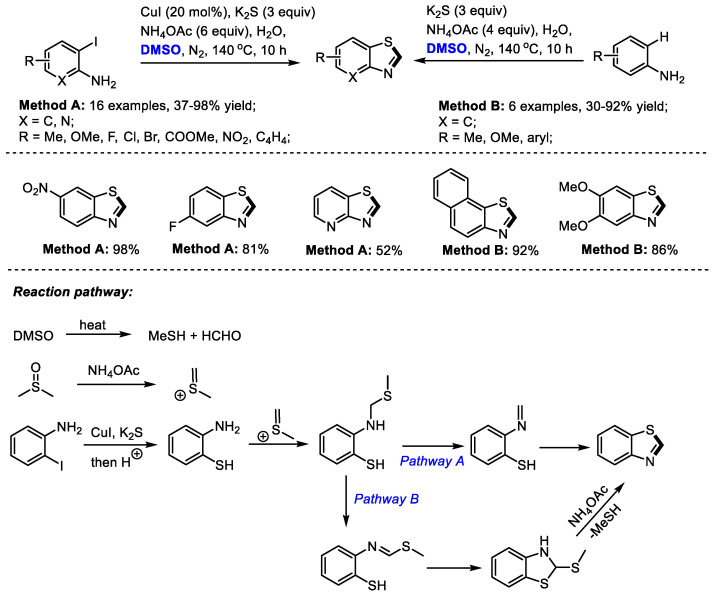
NH_4_OAc-mediated synthesis of benzothiazoles [27].

Alternatively, electron-rich aromatic amines can be used for the construction of 2-unsubstituted benzothiazoles in the absence of CuI (Method B: six examples, 30–92% yield). K_2_S served as the sulfur source and DMSO acted as the carbon source for both reaction systems (Scheme 13) [27].

### 2.7. Synthesis of Benzimidazole

**Scheme 14:**
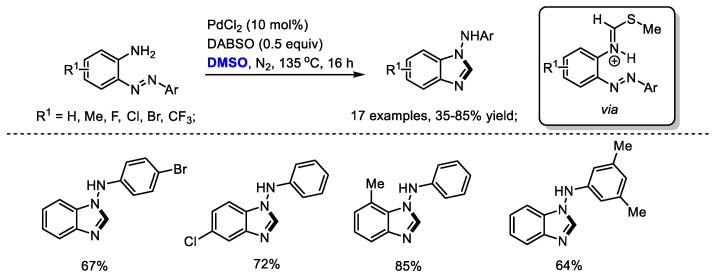
Pd-catalyzed synthesis of benzimidazoles [28].

By applying a strategy of a palladium-catalyzed oxidative formation of the key iminium intermediate, Cheng and coworkers prepared a variety of *N*-aryl-1*H*-benzo[*d*]imidazol-1-amines in moderate to good yields (17 examples, 35–85% yields). The 1,4-Diazabicyclo [2.2.2] octane bis(sulfur dioxide) adduct (DABSO) played dual roles in this reaction system, including the activation of DMSO to incorporate methine fragment into benzimidazoles and the oxidation of Pd(0) to Pd(II) (Scheme 14) [28].

**Scheme 15:**
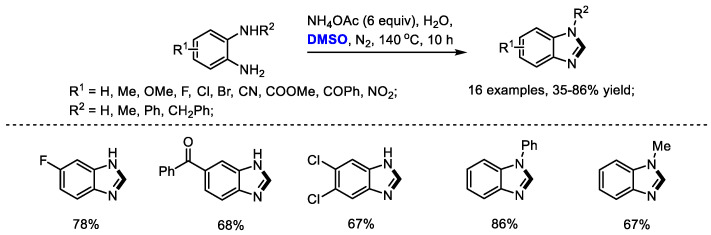
NH_4_OAc-mediated synthesis of benzimidazoles [27].

In addition, Zhu, Liang and Yu further extended their methination strategy to the preparation of benzimidazoles. A variety of 2-unsubstituted benzimidazoles were successfully constructed with *o*-phenylenediamines and dimethyl sulfoxide in the presence of ammonium acetate (16 examples, 35–86% yield). This process also featured good functional group compatibility and high efficiency (Scheme 15) [27].

### 2.8. Synthesis of Furan

**Scheme 16:**
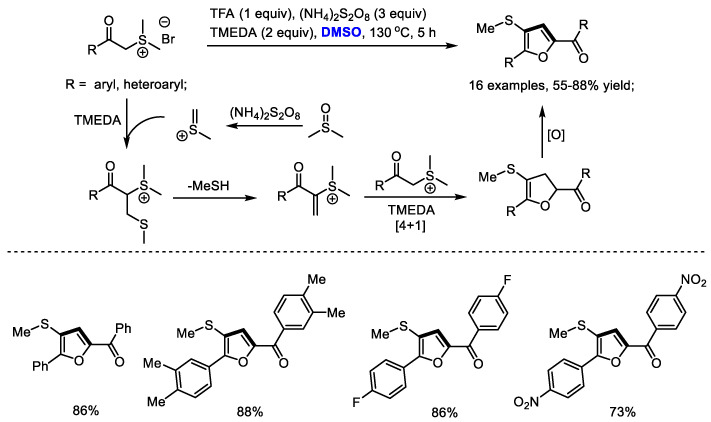
Synthesis of furans with stabilized sulfonium salts and DMSO [29].

Versatile sulfur ylides are capable of participating in various cycloadditions for synthesizing carbo- and heterocycles. Shu and Wu established a facile synthesis of multisubstituted furans with stabilized sulfonium salts and dimethyl sulfoxide (Scheme 16) [29]. α-Methylene sulfonium salt was formed as the key intermediate, which would undergo a [4 + 1] annulation with the sulfur ylide to yield dihydrofuran. A variety of aryl groups can be tolerable in this process (16 examples, 55–88% yields). Unfortunately, alkyl groups failed to produce the desired furans.

## 3. DMSO as the Source of CH_2_

Isoindolinone derivatives have displayed profound physiological and chemotherapeutic properties. For instance, *N*-substituted isoindolinone is present in a range of biologically interesting natural products and synthetic drugs such as triazol-isoindolinone (MGR-1 antagonist), deoxythalldomide (anti-tumor activity) and indoprofen (anti-inflammatory). Shi and coworkers reported a convenient synthesis of isoindolinones with 3,4,5-trimethoxybenzoic acid, amides and dimethyl sulfoxide through a Pummerer-type rearrangement. Various substituted sulfonamides and amides can be applicable in this process, allowing access to interesting *N*-substituted isoindolinones in moderate to good yields (31 examples, up to 85% yield). 

**Scheme 17:**
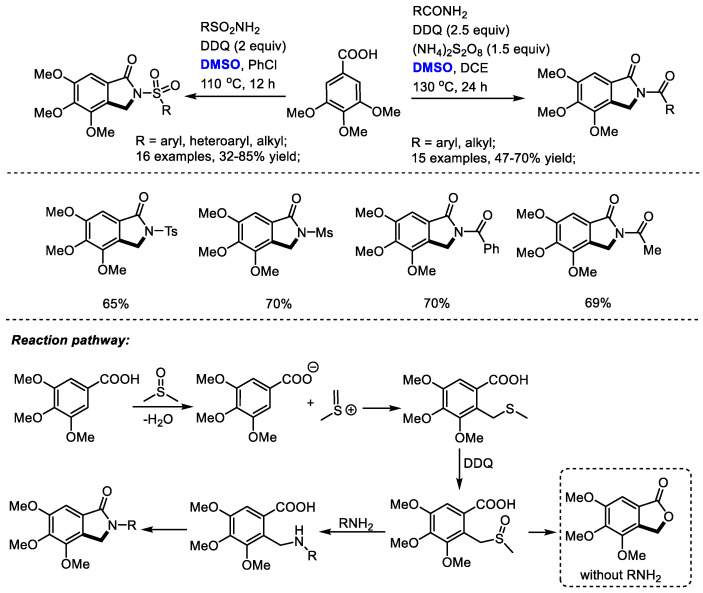
DMSO-based synthesis of isoindolinones [30].

An active species, methyl(methylene)sulfonium, was proposed to be generated from DMSO in the presence of acid. A subsequent electrophilic addition of methyl(methylene)sulfonium with 3,4,5-trimethoxybenzoic acid gave a thioether which would be oxidized by DDQ immediately. A following nucleophilic substitution of the sulfoxide with amide and a final lactamization yielded the isoindolinone product. Alternatively, the C–S bond cleavage may be followed by the formation of a C–O bond in the absence of amide yielding lactone 4,5,6-trimethoxyisobenzofuran-1(3*H*)-one (Scheme 17) [30].

**Scheme 18:**
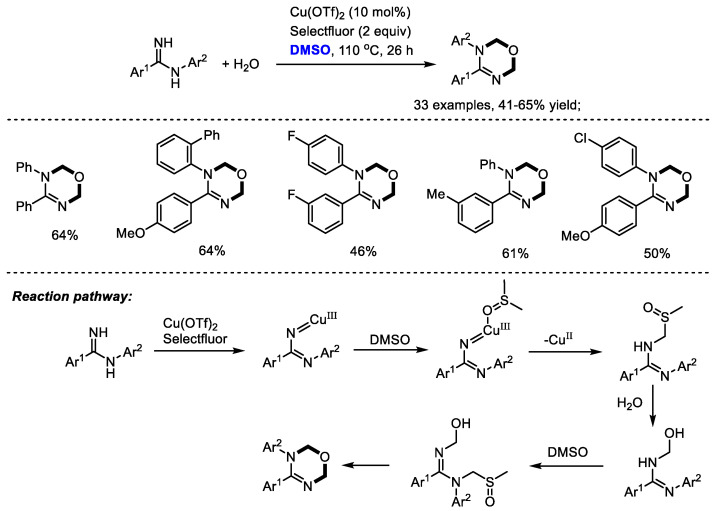
Cu-catalyzed synthesis of 1,3,5-oxadiazines [31].

Oxadiazines have been regarded as important scaffolds because of their wide occurrence in many biologically active compounds which exhibit cardiovascular, antitumor and insecticidal activities. The Ma group efficiently synthesized a vast number of 1,3,5-oxadiazines with amidines. Dimethyl sulfoxide was used as a dual carbon synthon and water was utilized as an oxygen donor (33 examples, 41–65% yield). The authors found that the positions of substituents on the aryl rings have no significant influence on the reaction yield, whereas the electronic nature apparently affected the yield. Unfortunately, heteroaryl-substituted amidines failed to yield the desired product. As proposed by the authors, a copper-nitrene intermediate would be generated in the presence of copper salts and selectfluor. The following coordination with DMSO and nitrene insertion in the C(*sp*3)-H of DMSO led to the formation of a sulfoxide intermediate. Further nucleophilic replacement with water, a similar insertion into DMSO, nucleophilic replacement with water, and annulation yielded the desired oxadiazine (Scheme 18) [31].

## 4. DMSO as the Source of Quaternary C

**Scheme 19:**
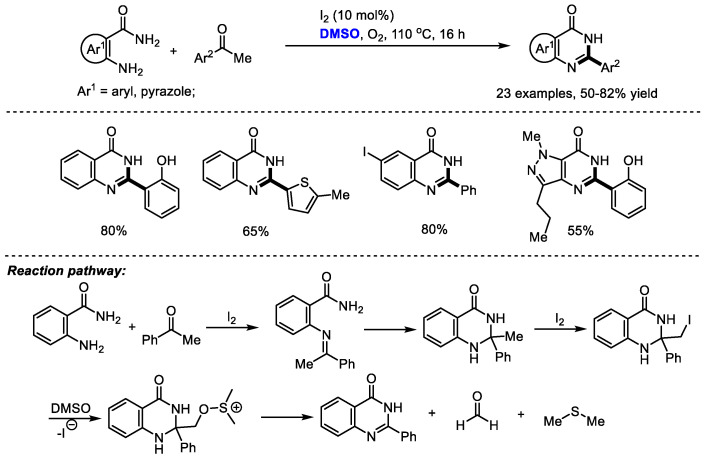
I_2_-catalyzed synthesis of quinazolinones [32].

Dimethyl sulfoxide can also be used as a carbon source by introducing a quaternary carbon into a heterocyclic molecule. Given the great importance of 2-aryl substituted quinazolinones such as the potent kinase inhibitor idelalisib as promising biologically active compounds, Vishwakarma and Bharate prepared a series of quinazolin-4(3*H*)-ones and pyrazolo [4,3-*d*]pyrimidi-7(6*H*)-ones via the iodine-catalyzed oxidative amination of the *sp*3 C–H bond. It was found that the quantity of iodine played a crucial role in the selectivity in this reaction (Scheme 19) [32]. When using a stochiometric amount of iodine, a 2-benzoyl substituted product was obtained as the major product by forming a glyoxal intermediate. A broad range of substituted acetophenones and 2-aminobenzamides have been proven to be tolerable in this process (23 examples, 50–82% yield). 

Additionally, pyrazolo-pyrimidiones can be produced readily by this protocol in moderate yields. The key steps of this reaction may be the iodination of 2-methyl-2-aryl-2,3-dihydroquinazolin-4(1*H*)-one intermediate and further fragmentation by the attack with DMSO to give quinazolinone, formaldehyde and dimethyl thioether. 

## 5. DMSO as the Source of CH and CH_3_

**Scheme 20:**
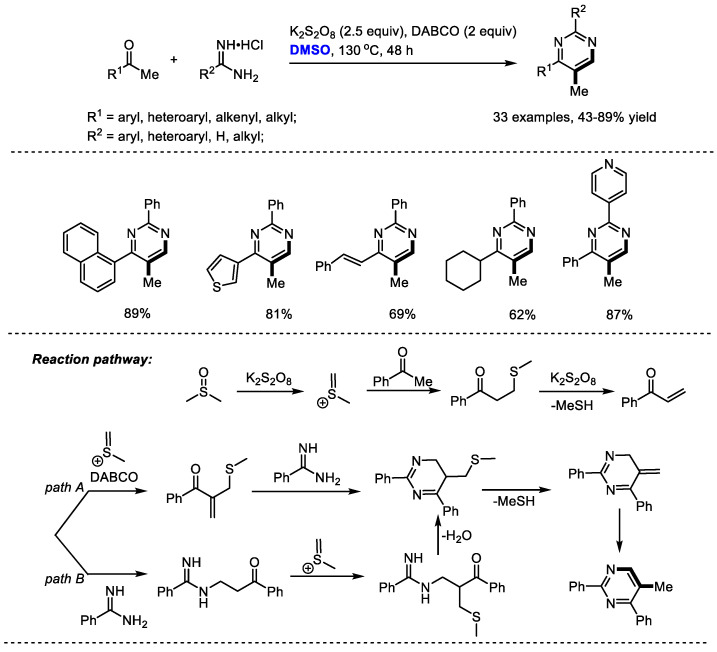
K_2_S_2_O_8_-mediated synthesis of pyrimidines [33].

DMSO has served as a dual synthon by introducing both CH and CH_3_ moieties into 5-methyl pyrimidine derivatives. In 2018, Wu and Jia developed a four-component oxidative annulation of methyl ketones, amidine hydrochlorides and dimethyl sulfoxide in the presence of K_2_S_2_O_8_ and DABCO (Scheme 20) [33]. Wide substrate scopes of both methyl ketones and amidines were observed in this process (R^1^ = aryl, heteroaryl, alkenyl, alkyl; R^2^ = aryl, heteroaryl, H, alkyl; 33 examples, 43–89% yield). 

A mechanistic study implied that an in situ-formed α,β-unsaturated enone, which may be generated from the addition of methyl ketone with methyl(methylene)sulfonium and a following elimination of MeSH, should be an important intermediate. Two possible routes might be involved in the formation of the annulated (methylthio)methyl substituted dihydropyrimidine intermediate. Sequential demethylthiolation and tautomerization gave the desired pyrimidine. K_2_S_2_O_8_ was employed as the activator of DMSO for generating an active methyl(methylene)sulfonium species.

## 6. DMSO as the Source of SCH_3_

**Scheme 21:**
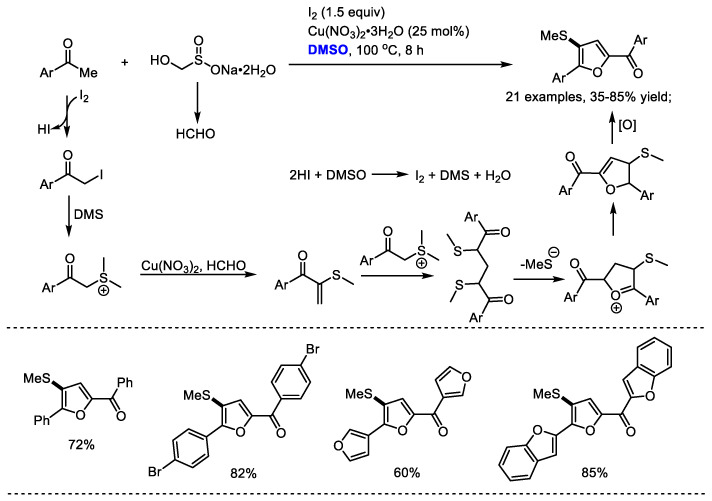
I_2_/Cu(NO_3_)_2_·3H_2_O promoted synthesis of furans [34].

Wu and Wu achieved an efficient I_2_/Cu(NO_3_)_2_·3H_2_O-promoted triple C(*sp*3)-H functionalization for the assembly of 2,4,5-trisubstituted furans in 2016 (Scheme 21) [34]. Rongalite (sodium hydroxymethanesulfinate) was employed as a C1 unit and DMSO was used as the source of SMe and the oxidant. This process was applicable to the preparation of a broad range of (2-acyl-4-methylthio-5-aryl)-furans (21 examples, 35–85% yield). 

A mechanistic study revealed that the in situ-formed dimethyl(phenacyl)-sulfonium iodine and formaldehyde were the key intermediates in this reaction. The formation of dimethyl(phenacyl)-sulfonium iodine was the probable way of introducing the SMe group into the final furan product. Copper nitrate hydrate was proposed as the catalyst for promoting the condensation of dimethyl(phenacyl)-sulfonium iodine with formaldehyde. It is worth noting that the obtained (2-acyl-4-methylthio-5-aryl) furans in this study are difficult to prepare by the documented procedures.

**Scheme 22:**
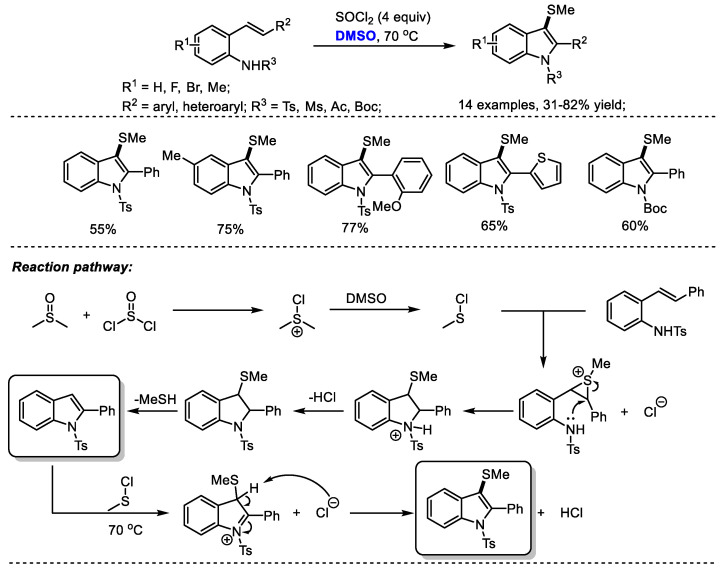
DMSO/SOCl_2_-promoted synthesis of 3-methylthioindoles [35].

A temperature-dependent switchable DMSO/SOCl_2_ promoted C(*sp*2)-H amination of 2-alkenylanilines for the synthesis of 3-unsubstituted indoles and 3-methylthioindoles was established by Du and coworkers (Scheme 22) [35]. 3-Unsubstistituted indoles were obtained at room temperature through intramolecular cyclization and elimination; while higher temperature (70 °C) yielded 3-methylindoles through further electrophilic methylthiolation. Given the wide occurrence of 3-methylthioindole moieties in pharmaceutically important molecules due to their interesting biological activities, the authors prepared a range of functionalized 3-methylthioindoles (14 examples, 31–82% yield). Various functional groups can be well tolerated at the positions of R^1^ (F, Br, Me), R^2^ (aryl, heteroaryl) and R^3^ (Ts, Ms, Ac, Boc). 

On the basis of their results, the authors proposed that the active species MeSCl would be generated in situ by the reaction of dimethyl sulfoxide with thionyl chloride. A MeSCl-triggered cyclization, the elimination of MeSH, electrophilic methylthiolation and aromatization delivered the desired 3-methylindoles.

**Scheme 23:**
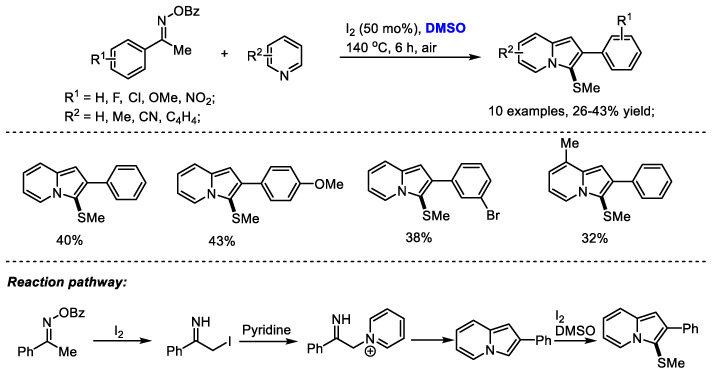
I_2_-promoted synthesis of imidazopyridines [36].

Imidazopyridine as a privileged framework is a nitrogen-bridged heterocycle widely present in biologically interesting compounds including Olprinone, Saripidem, Zolimidine, Zolpidem, Alpidem and Necopidem. The Maity group reached a molecular iodine-mediated facile synthesis of 3-sulfenylated imidazo[1,2-*a*]pyridines through the generation of iminyl radicals as active intermediates from oximes (Scheme 23) [36]. 2-Iodo-1-phenylethan-1-imine was generated through a radical pathway and then reacted with pyridine, yielding imidazopyridine. A further methylthiolation in the presence of dimethyl sulfoxide and molecular iodine yielded the desired product.

## 7. DMSO as the Source of CH_2_SCH_3_

**Scheme 24:**
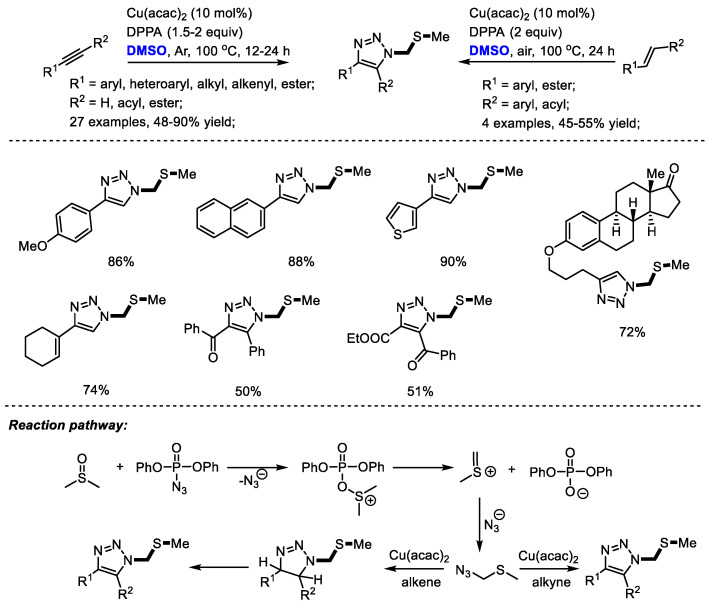
Cu-catalyzed synthesis of sulfur-containing triazoles [37].

Sulfur-containing triazole as a class of pharmaceutically interesting agents may show mPGES-1 inhibition activity and anti-inflammatory activity. The Jiao group reached a novel copper-catalyzed synthesis of sulfur-containing triazoles with DPPA (diphenylphosphoryl azide), DMSO and alkynes/alkenes. Aromatic, aliphatic, and even internal alkynes could all be compatible in this process providing corresponding triazoles efficiently (27 examples, 48–90% yield) [37]. α,β-Unsaturated ketones can also be used as suitable substrates for synthesizing triazoles in moderate yields with high regioselectivity (four examples, 45–55% yield). Notably, the late-stage modification of natural product derivatives such as estrone and estradiol has been achieved successfully by the incorporation of sulfur-containing moiety. A key intermediate, (azidomethyl) (methyl)sulfane, was suggested to be yielded by the reaction of in situ-generated methyl(methylene)sulfonium and azide ion. A further click reaction yielded the functionalized triazoles (Scheme 24).

## 8. DMSO as the Source of CH_2_ and CH_2_SCH_3_

**Scheme 25:**
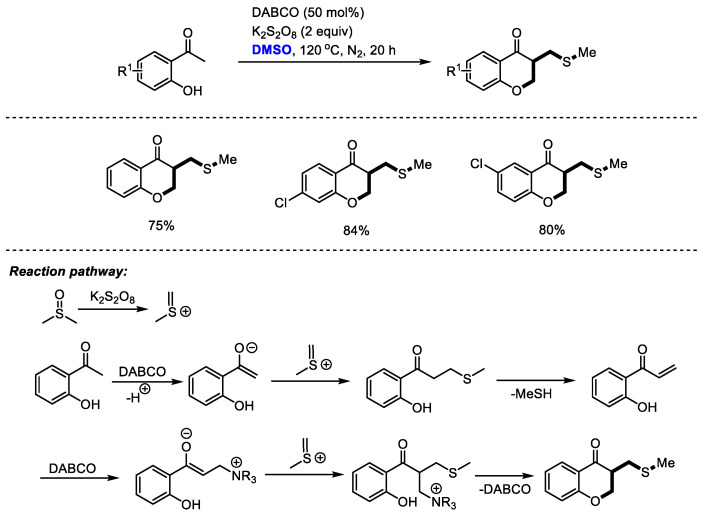
DABCO-promoted construction of chroman-4-ones [38].

The introduction of both CH_2_ and CH_2_SCH_3_ moieties was realized stepwise through the efficient transformations of methyl(methylene)sulfonium intermediate. Guo and coworkers achieved an efficient DABCO-promoted construction of chroman-4-one derivatives with *o*-hydroxyacetophenones and dimethyl sulfoxide (Scheme 25) [38]. As proposed by the authors, methyl(methylene)sulfonium cation would be generated firstly by activating DMSO with K_2_S_2_O_8_. Methylthiomethylenation and a following demethylthiolation resulted in the formation of enone. A further α-methylthiomethylenation and a final annulation led to the formation of the desired chroman-4-one. 

## 9. DMSO as the Source of O

**Scheme 26:**
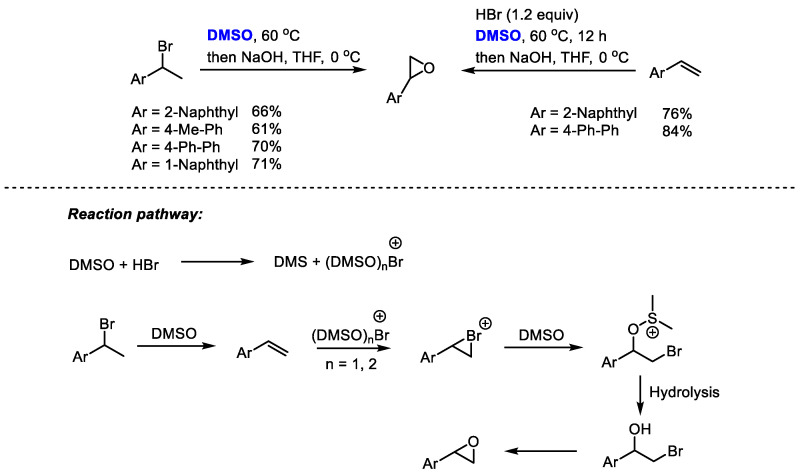
DMSO-based synthesis of epoxides [39].

The HBr/DMSO reaction system has been extensively used for the functionalization of alkenes and arenes through bromination and hydroxybromination. Additionally, HBr/DMSO can also be utilized for preparing epoxides through a one-pot procedure. Jiao and coworkers realized a one-pot conversion of secondary bromides or olefins into epoxides employing dimethyl sulfoxide as a cheap oxidant and oxygen source as well as a solvent (Scheme 26) [39]. 

(DMSO)_n_Br^+^ was proposed to be the key active brominating species in this reaction system. The electrophilic addition of Br^+^ with alkene yielded bromonium intermediate, which was followed by a nucleophilic attack of DMSO and hydrolysis. The epoxidation of bromohydrin yielded the final product. This process features easy operability, readily available and cheap reagents and simple reaction conditions.

## 10. DMSO as the Oxidant

In DMSO-based reaction systems for heterocycle synthesis, DMSO can be used as a crucial oxidant through a Kornblum oxidation, the oxidative formation of disulfides, and oxidative aromatization.

**Scheme 27:**
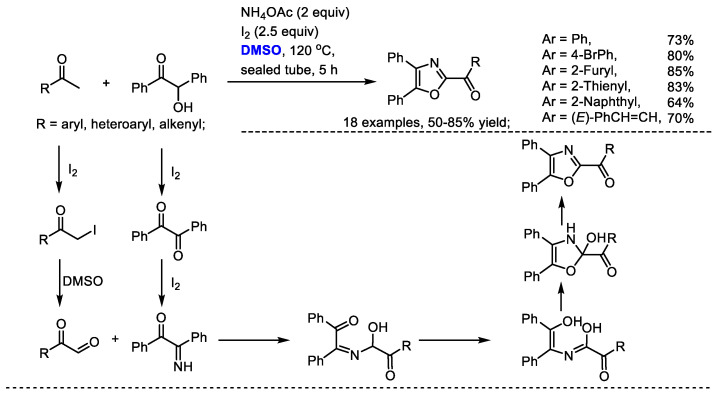
DMSO-based synthesis of oxazoles [40].

Oxazole derivatives are biologically interesting compounds as well as being valuable building blocks. Wu and coworkers realized a convergent integration of two self-assembly domino sequences for synthesizing oxazole derivatives with methyl ketones, benzoins and ammonium acetate (Scheme 27) [40]. Aldehydes bearing aryl, heteroaryl and alkenyl groups can be compatible in this process (18 examples, 50–85% yield). This methodology features simple starting materials, mild conditions and easy operation. Arylglyoxal was obtained through the iodination of aryl methyl ketone and a following Kornblum oxidation in the presence of DMSO. Intramolecular proton-transfer of the adduct of 2-imino-1,2-diphenylethan-1-one with arylglyoxal and a final dehydration yielded the desired oxazole.

**Scheme 28:**
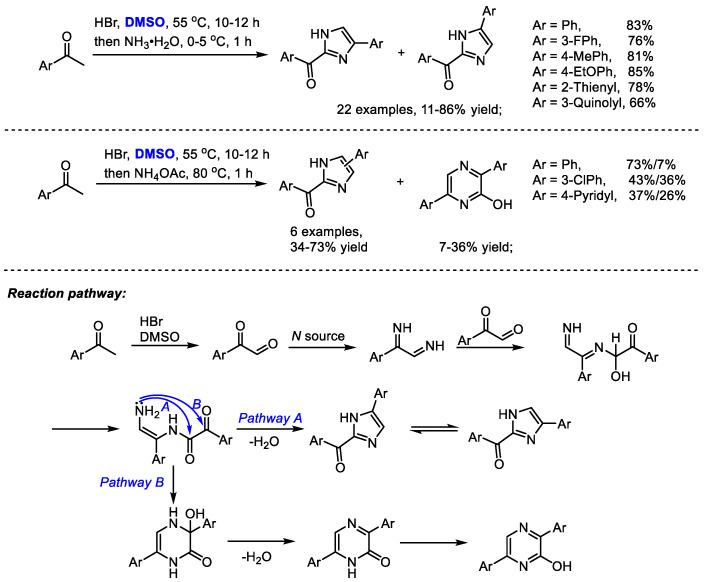
Synthesis of imidazoles in the presence of HBr and DMSO [41].

The Dai group established a one-pot synthesis of 2-aroyl-(4 or 5)-aryl-1*H*-imidazoles with aryl methyl ketones, hydrobromic acid, dimethyl sulfoxide and an ammonium source in 2014 (Scheme 28) [41]. A series of imidazoles were prepared efficiently (22 examples, 11–86% yield). The reaction was proposed to undergo a HBr/DMSO mediated α-bromination, Kornblum oxidation and Debus–Radziszewski imidazole synthesis. Interestingly, pyrazines can be obtained when switching to ammonium acetate as a nitrogen source. This process features easily available materials, mild reaction conditions and easy operation. DMSO served as the oxidant in the Kornblum oxidation.

**Scheme 29:**
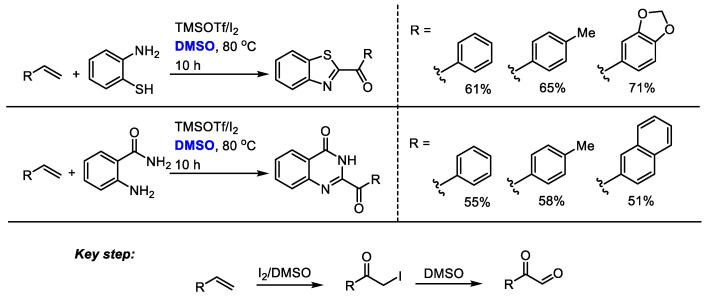
I_2_/TMSOTf mediated synthesis of benzothiazoles and quinazolines [42].

Shah and coworkers established a metal-free oxidative amidation of terminal alkanes in the presence of molecular iodine and dimethyl sulfoxide in 2015 (Scheme 29) [42]. The key steps for the synthesis of benzothiazoles and quinazolines were the I_2_/DMSO-mediated formation of α-iodoacetophenone and a following Kornblum oxidation to give a 2-oxoaldehyde. Corresponding benzothiazoles and quinazolines can be formed in moderate to good yields through subsequent condensation, annulation and aromatization.

**Scheme 30:**
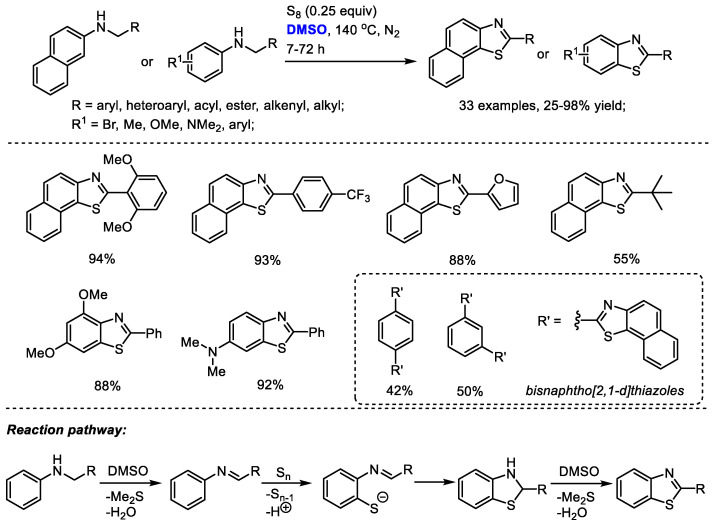
DMSO-involved synthesis of benzothiazoles and naphtho [2,1-*d*]yhiazoles [43].

The DMSO-involved oxidative formation of imine and oxidative aromatization can be employed for heterocycle synthesis. Deng and Liang reported an efficient protocol for the facile synthesis of benzothiazoles and naphtho [2,1-*d*]yhiazoles with *N*-substituted arylamines and elemental sulfur (Scheme 30) [43]. Dimethyl sulfoxide was used as the oxidizing reagent for the oxidation of *N*-substituted arylamine into imine and the final oxidative aromatization as well as the solvent. The electrophilic addition of the in situ-generated imine with elemental sulfur (S_8_) was followed by an elimination of elemental sulfur (S_7_) and proton. A subsequent intramolecular cyclization yielded thiazoline intermediate and a DMSO-mediated oxidative aromatization delivered the desired product. This approach was capable of producing a broad range of highly functionalized benzothiazoles and naphtho [2,1-*d*]yhiazoles (33 examples, up to 98% yield).

**Scheme 31:**
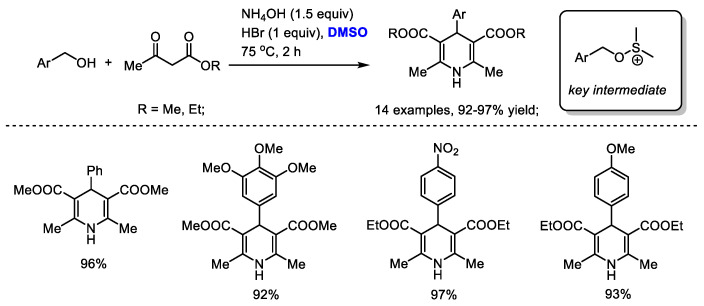
DMSO-involved synthesis of Hantzsch 1,4-dihydropyridines [44].

A facile synthesis of Hantzsch 1,4-dihydropyridines utilizing a HBr/DMSO reaction system was established by Ranjbar and coworker (Scheme 31) [44]. A variety of substituted benzyl alcohols could be compatible to produce 1,4-dihydropyridines in excellent yields (14 examples, 92–97% yield). A (benzyloxy)dimethylsulfonium was suggested as a key intermediate to undergo a nucleophilic substitution reaction with a 1,3-dicarbonyl derivative. The in situ-formed bromodimethylsulfonium bromide may also be responsible for the oxidative generation of a conjugated imine for further electrocyclization.

**Scheme 32:**
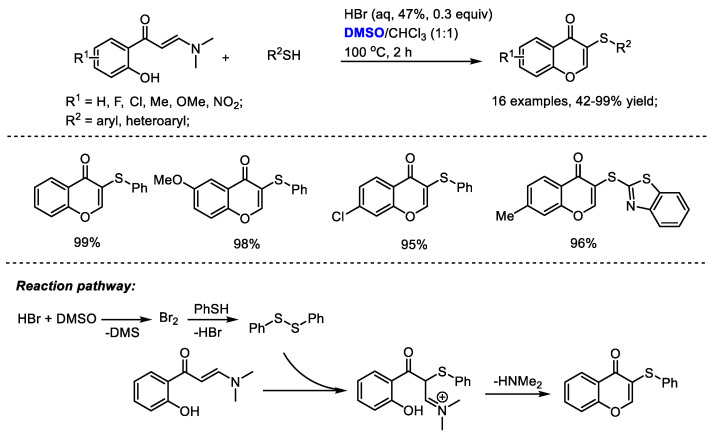
HBr-catalyzed synthesis of sulfenylated chromones [45].

Disulfide can be formed in the presence of in situ-generated molecular bromine. The Maddani group developed an efficient catalytic construction of sulfenylated chromones in the presence of aqueous HBr and DMSO (Scheme 32) [45]. A range of substituted enaminones and aryl thiols can be compatible in this process producing excellent yields in most cases (16 examples, 42–99% yield). DMSO served as an oxidizing agent responsible for the in-situ generation of molecular bromine. Disulfide was yielded from thiol under the catalysis of bromine. A subsequent electrophilic addition of *o*-hydroxyphenyl *N*,*N*-dimethyl enaminone with disulfide delivered sulfenylated chromone.

**Scheme 33:**
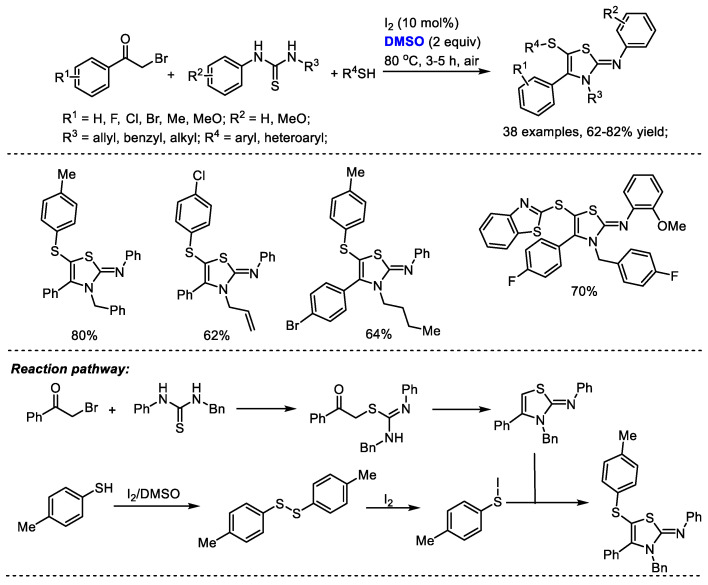
I_2_-catalyzed synthesis of 5-sulfenyl-2-iminothiazolines [46].

Disulfide formation can be realized under a I_2_/DMSO reaction system for further heterocycle construction. The Pramanik group reached a one-pot four-component construction of 5-sulfenyl-2-iminothiazolines through cross-dehydrogenative C–S coupling in the presence of I_2_ and DMSO (Scheme 33) [46]. A large number of 5-sulfenyl-2-iminothiazoline derivatives were assembled efficiently with various arylacyl bromides, thioureas and aromatic and heterocyclic thiols (38 examples, 62–82% yield). Significant features of this methodology include metal-free open air reaction conditions, a broad substrate scope, high efficiency and good reaction yields. A disulfide intermediate can be generated by the treatment of thiol with iodine and dimethyl sulfoxide. An active aryl hypoiodothioite species was then generated in the presence of iodine which would undergo electrophilic sulfenylation. DMSO served as the oxidizing agent in this process.

**Scheme 34:**
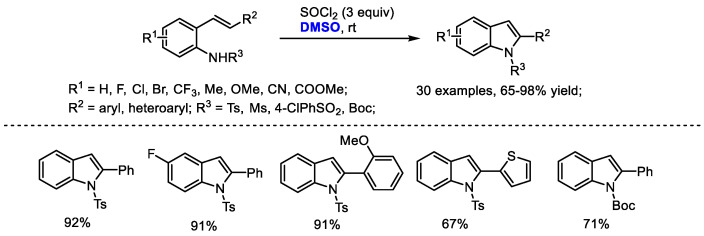
DMSO/SOCl_2_-mediated synthesis of indoles [35].

Substituted indoles were prepared through a DMSO/SOCl_2_-mediated oxidative C(*sp*2)-H amination strategy by Du and coworkers (Scheme 34) [35]. The intramolecular annulation of 2-alkenylanilines can be reached by the combination of DMSO and SOCl_2_ at room temperature. This reaction has been proved to be quite general allowing the facile introduction of functional groups into the indole skeleton (30 examples, 65–98% yield). The authors proposed that an active MeSCl intermediate generated from DMSO and thionyl chloride would initialize an intramolecular cyclization and a following elimination led to the formation of indole (Scheme 22).

## 11. DMSO-Based Heterocycle Synthesis through Halogenation

**Scheme 35:**
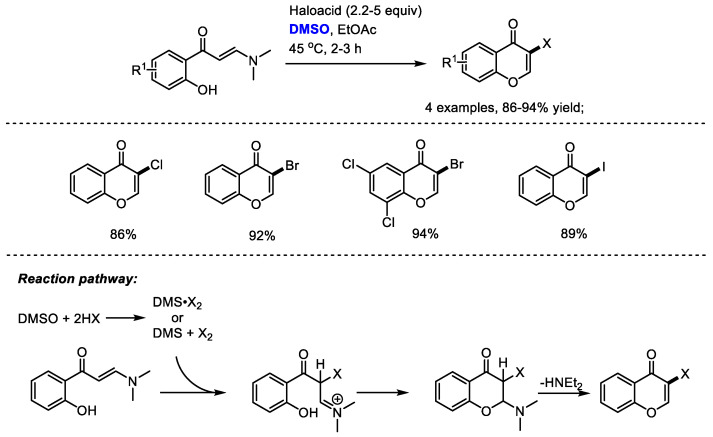
DMSO-involved synthesis of halogenated chromenones [47].

Besides the oxidative transformations such as the halogenation of arenes, dihalogenation of arenes, benzylic oxidation, oxidation of ketones, the haloacid/DMSO reaction system can also be used for the direct construction of heterocycles. Maddani and coworkers disclosed a metal-free and efficient synthesis of halogenated chromenone derivatives with (*E*)-3-(dimethylamino)-1-(2-hydroxyphenyl)prop-2-en-1-ones, haloacids and dimethyl sulfoxide (Scheme 35) [47]. 

As suggested by the authors, haloacid would react with dimethyl sulfoxide, resulting in the generation of DMS·X_2_. The halogenation of enaminone and subsequent cyclization led to the formation of a chromone product. While direct halogenated products would be yielded when using *N*-aryl enaminones without the phenolic hydroxy group.

## 12. Conclusions and Outlook

In conclusion, various heterocycles including pyridines, quinolines, phenanthridines, pyrimidines, quinazolines, benzothiazoles, benzimidazoles, indoles, imidazoles, triazoles, imidazopyridines, oxazoles, oxadiazines, chromenones, and furans were successfully constructed with acyclic starting materials under dimethyl sulfoxide-based reaction systems. Dimethyl sulfoxide was utilized efficiently in these direct syntheses of *N*- and *O*- heterocycle as a carbon source, oxygen source, and sulfur source as well as an oxidizing agent. A variety of fragments and functional groups, such as C, CH, CH_2_, CH_3_, SCH_3_, CH_2_SCH_3_, and O, can be introduced into the target molecules. Methyl(methylene)sulfonium, chlorodimethylsulfonium, bromodimethylsulfonium, molecular bromine, arylglyoxal, and imine have been suggested as the key active species enabling these transformations. As a synthon for methodology development, DMSO has many advantages: low toxicity, easy accessibility, good compatibility in many reaction systems, high flexibility in its reaction design and diverse choices of activator. 

However, to further explore potential applications of DMSO in the synthesis of heterocyclic molecules as well as other useful compounds, more efforts should be made in this research field. Firstly, novel effective activators for DMSO should be developed in order to create new reaction pathways and/or active species. Since Bronsted acids, Lewis acids, Bronsted bases, oxidants, acyl halides, and acyl anhydrides, etc. all can be used for the activation of DMSO, there are many options for developing different activating pathways. Secondly, the combination of other powerful catalysts or catalytic systems with DMSO-derived active species would be another possible way to realize a new chemistry. A logical reaction design could be inspired by synergistic catalysis and bifunctional catalysis reaction systems. Other controlling parameters including photoinduced and electrochemical ones on reaction systems can also be added into the methodology development. Thirdly, the DMSO-involved modification of already established heterocyclic architectures may be a rapid and easy way of producing highly functionalized molecules. There are two possible extensions for this strategy: a one-pot or cascade reaction system design for functionalizing the in situ-generated heterocycles, and a modification reaction design using heterocyclic substrates. For instance, as proved by the methods discussed in this review, temperature-controlled or substrate-controlled approaches were able to produce different types of functionalized molecules.

## Data Availability

Not applicable.
